# The Two Adjuncts of Sanatorium Treatment

**Published:** 1913-08-23

**Authors:** 


*To the Editor of* The Hospital.

### The Two Adjuncts of Sanatorium Treatment.

*To the Editor of* The Hospital.

Sir,—The article in The Hospital last week on "Tuberculosis Mistakes" touched upon the importance of the points which have been efficiently dealt with by the authorities here.

It has come to be very clearly recognised that the sanatorium treatment of tuberculosis is in itself only a link in a chain, unless accompanied by adequate provision for the dependants of the patient while undergoing the sanatorium course, and afterwards by an efficient after-care system.

It may be of interest in view of these considerations to give a short account of a sanatorium which for a very long time was the only one having a definite after-care branch in connection with it, worked by a special committee whose object it is to supervise and keep in touch with all discharges. An official visits, from time to time, the patients whilst at the sanatorium, thus enabling himself to form opinions about each one's suitability for following more healthy occupation, or for emigration. Every inducement is made also to move them out of the congested districts where they may live, as they are all drawn from the poorer parts of London. The care of the dependants, also, is dealt with by this committee, by allowance made to them weekly as necessity calls, or, where the patient is the mother, a woman paid for to go in to look after the children and home. The peace of mind thus assured to the patient away from his or her home is a very real aid to the treatment and progress while in the sanatorium. From experience one knows only too well how much the reverse can hinder and retard the recovery.

The sanatorium referred to is maintained philanthropically, and is more or less a private one, run upon lines of its own, and the ten years of its existence have gone to show clearly that without the after-care branch, as well as the system of provision for the families, its benefits would be of little lasting good. These expenses naturally add greatly to the maintenance of the sanatorium, but are reckoned as a necessary part, without which, as far as experience *here* goes, the rest would be waste of money.—Yours faithfully,

Sanatorium Officer.

August 18, 1913.

